# Voice of the Clinician: the case of an Australian health system

**DOI:** 10.1108/JHOM-05-2017-0113

**Published:** 2017-09-18

**Authors:** Mark J. Lock, Amber L. Stephenson, Jill Branford, Jonathan Roche, Marissa S. Edwards, Kathleen Ryan

**Affiliations:** 1 Committix Pty Ltd, Newcastle, Australia; 2 School of Business, Clarkson University, Schenectady, New York, USA; 3 Mid North Coast Local Health District, Coffs Harbour, Australia; 4 Clinical Governance Unit, Mid North Coast Local Health District, Coffs Harbour, Australia; 5 UQ Business School, The University of Queensland, Brisbane, Australia

**Keywords:** Australia, Communication, Decision making, Governance, Networks

## Abstract

**Purpose:**

The Voice of the Clinician project commenced during an era when practitioner burnout, dissatisfaction, and turnover became an increasingly global health workforce concern. One key problem is clinical staff not being empowered to voice their concerns to decision-makers, as was found in this case study of an Australian public health organization. The following research question informed the present study: What is a better committee system for clinician engagement in decision-making processes? The paper aims to discuss this issue.

**Design/methodology/approach:**

The Mid North Coast Local Health District in New South Wales aspired to improve engagement between frontline clinicians and decision-makers. Social network analysis methods and mathematical modeling were used in the discovery of how committees are connected to each other and subsequently to other committee members.

**Findings:**

This effort uncovered a hidden organizational architecture of 323 committees of 926 members which overall cost 84,729 person hours and AUD$2.923 million per annum. Furthermore, frontline clinicians were located far from centers of influence, just 37 percent of committees had terms of reference, and clinicians reported that meeting agendas were not being met.

**Practical implications:**

In response to the findings, a technological platform was created so that the board of directors could visually see all the committees and the connections between them, thus creating ways to further improve communication, transparency of process, and – ultimately – clinician engagement.

**Originality/value:**

The breakthrough idea is that all organizational meetings can be seen as a system of engagement and should be analyzed to determine and describe the points and pathways where clinician voice is blocked.

## Background

In early 2015, the Mid North Coast Local Health District (MNCLHD), a part of the New South Wales (NSW) Health System of Australia, determined that clinical engagement was lacking throughout the organization (NSW Ministry of Health, 2016). Specifically, clinicians within the organization felt that they were not being listened to, despite the fact that they were in direct contact with patients and best placed to inform decision-makers of patient needs. This loss of clinician voice, or the “provision of information intended to improve organizational functioning to someone inside an organization with the perceived authority to act” (Detert and Burris, 2007, p. 869), was recognized as a potential threat to the organization and a phenomenon that merited immediate attention.

In an effort to address these concerns, leaders at MNCLHD partnered with a research team to develop and execute the Voice of the Clinician (VotC) project. The VotC project was designed to address the lack of engagement between frontline clinicians and organizational decision-makers through the adaptation of an existing technology developed for social network research that determined and described the clinician engagement landscape with the view to, ultimately, track committee interactions. Social network theory (Butts, 2008; Luke and Harris, 2007; Wholey *et al.*, 2009) provided the underlying framework and tool for analysis of knowledge flows in a complex organization such as a hospital or health system setting. Furthermore, the VotC project examined the connectivity and relatedness of clinicians and decision-makers at the MNCLHD.

For context, the MNCLHD encompasses more than 3,000 clinical staff providing services to 212,193 residents living in rural and coastal settings over a geographical area of 4,376 square miles of NSW, which is the largest state in Australia (NSW Ministry of Health, 2015). These services are delivered in seven public hospitals, ten community health centers, and several specific facilities including oral health clinics, drug and alcohol services, and sexual health services. The MNCLHD definition of clinicians, and therefore the definition adopted for this study, includes all health professional staff – medical doctors, nurses, dentists, dietitians, podiatrists, occupational therapists, etc. Given the geographical reach and scope of the service population, providing a realistic solution for the MNCLHD organization was a critical outcome of the present research.

As such, this paper presents the first-year experiences of the VotC project and describes how the project leaders adapted a new, innovative technology to visually “map” the voice of clinicians. This mapping schema further permits health leaders to determine the distance of clinician voice from decision-makers. Using mathematical modeling, committee system mapping, and an organization-wide committee audit as baseline information, this paper introduces and discusses the value of technological intervention known as the Clinician Voice Hub (CVH). Through the present study, this paper shows that the CVH has potential for improving clinician engagement, ensuring robust governance mechanisms, and improving organizational efficiency in knowledge gathering for decision-making processes.

But perhaps the biggest contribution of this innovation lies in the unique way that the VotC project examines organizational governance. For the first time, the CEO and the board of the organization can see all committees and how they are linked, identify systemic barriers preventing effective committee operation, and further develop performance indicators (e.g. staff time, attendance rates, salary cost, completed terms of reference (ToR)). Specifically, the project renders “visible” otherwise hidden organizational architecture of committee meetings, takes a structural perspective through the use of social network analysis of staff engagement as opposed to organizational surveys, and applies an interactive technological knowledge platform for the MNCLHD. The implications of the project for clinician engagement are multifaceted. Any clinician can log into the cloud-based software to see all of the organizational committees, membership, and update their details for their own involvement. Clinicians can send and receive information through the software, using social media accounts, to other committee members that they identify through the interlock visualization in an effort to engage in dialogue about a relevant clinical issue. Access to this technology is sharply divergent from currently accepted practice whereby committee information is hoarded on local PC hard drives and available to only a privileged few. Instead, this software takes committee information off of the localized computer hard drive and into the cloud where it is accessible, measurable, modifiable, and invites a culture of transparency which further empowers clinicians clinician.

This project therefore, offered immediate translation of research-to-practice for the focal organization. In effect, it provides a baseline understanding of the internal organizational decision-making system. And, though one health system was the focus of the present study, replication efforts would be straight-forward to execute across a variety of health organization types.

## Statement of the problem: governance and engagement

In a complex organization, and particularly in healthcare, the internal decision-making systems are inextricably linked to governance structures (Kalita and Mondal, 2012; Tihanyi *et al.*, 2014). Governance generally refers to how organizations are controlled (Tihanyi *et al.*, 2014). Kalita and Mondal (2012) explain governance as “the way resources are allocated, the way decisions are made and the accountability mechanisms which are put in place” (p. 759). The importance of effective governance has reached national priority status such that Australia’s National Safety and Quality Health Service Standards (see Greenfield *et al.*, 2015) identify corporate governance (standard 1 – governance for safety and quality in health service organizations) as important to improving the performance of the organization for providing safe and quality services to patients. However, much of the existing literature examining governance has still only “primarily dealt with decision making by board and senior managers” (Tihanyi *et al.*, 2014, p. 1535).

While it is understandable why some scholars might limit the focus of governance research to top levels of the organization, it also neglects to understand governance in a holistic manner. Bluntly, when considering levels of governance, there is a long distance between the ceiling (board of directors) and the floor (clinician and patient interactions) of organizations. Ultimately, these prominent views in the literature stem from macro-financial perspectives thus discounting the invisible decision-making processes that take place in the space between the floor and the ceiling (Tihanyi *et al.*, 2014). For example, clinicians bring their concerns to the agenda of team meetings, the outcomes of which are then communicated up-the-line to higher level authority committees, the outcomes of which may reach the executive committee and then the board of directors. This flow represents a governance chain.

To capture this exchange, and instead of offering a common expression of governance by focusing on the board, the VotC project contributes to the extant literature as it measures clinician voice and strategic location of voice through the vehicle of committees, which are essential to the organizational architecture of decision-making processes. By using this approach, not only does the VotC project capture those concerns at the top levels of organizational governance but it also includes those governance mechanisms that influence clinician engagement. In this information communication network, inefficient structures prevent timely transfer of clinical issues from the clinicians up to the board. As Detert and Edmondson (2011) state, “It is the lack of timely input-from those who have information they believe is worth contributing, to those with the power to act-that especially hampers organizational learning” (p. 462). In addition to hampering organizational learning, such inefficiencies and hindrances of voice have also been linked to reducing clinician engagement (Swensen *et al.*, 2016).

Recently, clinician engagement has received a non-trivial amount of attention in the healthcare management landscape. This is not surprising as clinician engagement is often associated with outcomes such as patient safety (Swensen *et al.*, 2016), mortality and infection rates (Hewison *et al.*, 2013), quality of services rendered (Shanafelt *et al.*, 2012; Swensen *et al.*, 2016), and overall organizational effectiveness (Bonias *et al.*, 2012; Harter *et al.*, 2013). However, and despite the noted benefits, in a 2013 survey conducted within the NSW health system, the results showed that 66 percent of staff were “not being engaged” (Mid North Coast Local Health District, 2016).

One of the largest impediments that hinder an organization’s ability to achieve a fully engaged workforce is ineffective communication (Hewison *et al.*, 2013). Considering governance, and in terms of barriers to communication and thereby engagement, one obstacle includes the clinicians’ ideas for improvement not reaching the organizational decision-makers. As noted by Cohn (2015), “engagement drops when physicians feel that their questions aren’t answered and their frustrations aren’t being heard” (p. 60). This can occur through the top-down hierarchies of traditional healthcare management structures (Toussaint, 2016), through supervisor-supervisee interaction patterns stifling employee voice (Detert and Burris, 2007; Detert *et al.*, 2013; Detert and Edmondson, 2011; Detert and Trevino, 2010), or through inefficient meetings (Allen *et al*., 2016; Lehmann-Willenbrock *et al*., 2016). As an alternative, Sears (2011) explained that listening to the clinical perspective is one of the most effective ways to engage physicians and build a trusting relationship. For example, Toussaint (2016) called for changes to the typical, and arguably stale, twentieth century management system of top-down authoritarian approaches to more empowering mechanisms like open-ended status meetings. Similarly, the literature examining employee voice suggests openness and direct voice flows from the employee to the leader relate to the unit’s effectiveness and subordinate’s feelings of psychological safety (Detert and Burris, 2007; Detert *et al.*, 2013). And lastly, productive meeting behaviors have been shown to improve engagement and create satisfaction (Allen *et al.*, 2016) while counterproductive meeting behaviors correlate with exhaustion (Lehmann-Willenbrock *et al.*, 2016).

However, just as there is limited research on the tracking and measuring of the clinician voice, equally minimal attention has been paid to committee structures or meetings as a way to foster engagement. Despite the incredible amount of time and cost commitment spent by staff in meeting, it is noted by Allen *et al.* (2015) “Astonishingly, however, a scientific look at meetings as a focal topic remains largely elusive” (p. 4). This is surprising, as patients interact with clinicians who then communicate issues to internal organizational committees. These committees are essential structures of an organization’s governance processes as the knowledge garnered through them should feed into the quality and safety of systems promoting improvements in quality of care. However, there are barriers to this patient-clinician-committee-organization-service-patient pathway. For instance, the multitude of service occasions made by diverse clinician groups who report to many line managers that further feed issues upward to committees increases the probability that the clinician’s voice will be lost.

As suggested, if the clinician’s voice is lost, there is a much higher chance that they will become disengaged (see Detert *et al.*, 2013). When a clinician is not engaged, they are more susceptible to burnout (Graber *et al.*, 2008; Gregory and Menser, 2015; Okie, 2008; Thanacoody *et al.*, 2009), stress (Kuusio *et al.*, 2012; Williams *et al.*, 2002), dissatisfaction and – ultimately – turnover (Barney, 2002; Sadatsafavi *et al.*, 2015; Zuger, 2004). Therefore, it is necessary to gain a fuller understanding of why clinician voice is not being heard and to further develop an evidence-based mechanism of improving efficiency of organizational knowledge gathering processes from staff. This is particularly imperative as “optimal decision-making happens when information regarding decisions is collected at the local level” (Harter *et al.*, 2013, p. 3). Consequently, developing a committee system for mapping, measuring, monitoring, and evaluating system-wide connections and barriers therein aids in developing strategies to improve communication efficiency, transparency, and accountability. These are three important dimensions in healthcare governance (Barbazza and Tello, 2014) but also take into consideration how governance structures can influence clinician engagement.

## Methodology

The VotC project employed several approaches to better understand the current state of practice at MNCLHD and subsequently why clinician voice was not reaching the decision-makers. The project began with a committee mapping methodology through a newly designed technological innovation known as the CVH. The CVH is a centralized committee knowledge management hub based on an online database linked to a coded cloud-based software package which provides committee effectiveness and efficiency information to all MNCLHD clinicians through an engaging and interactive web interface. Through the CVH, clinicians can “see” how all committees and the connections from them are linked into the decision-making structure (a hierarchical perspective) and as interlinked into a knowledge diffusion network (a network perspective). The method is derived from the field of social network analysis, which is a mathematical way of describing and modeling various types of networks that occur in society (Butts, 2008; Luke, and Harris, 2007; Wholey *et al.*, 2009).

### Hierarchy and interlocked data

Collecting social network data requires defining the actual links among committees, and in the health system committees are linked in two ways – hierarchical and interlocked. The first data collection method determined the hierarchical links from subordinate to superordinate committees, for example the senior executive team of the MNCLHD reports to the board of directors. This formal reporting link data are often noted in the committee by-laws or charters or ToR. One member of the research team was tasked with the responsibility of investigating clinician engagement in the MNCLHD. This individual employed a link-trace snowball technique necessary for collecting social network data where the boundary of the network is unknown and formal information – published ToR – do not exist (Butts, 2008). This step began with the known committees published on the website of the MNCLHD as found by entering the search term “committee” into the website search box. Resulting committee information (ToR, membership) were entered into the CVH database.

The second data collection method collected the names of committee members, some of whom are members of other committees, and thus interlock different committees (Lamb *et al.*, 2016). This second step used the membership lists from the first step to seed in-person follow-up of committees’ secretariats to identify unpublished committees, and data were entered into the CVH. The individual collecting the data ensured the correct links existed between subordinate and superordinate committees by verbal corroboration from the committees’ secretariats. Data saturation was reached when no new committees were discovered.

### Data visualization

The two-mode network data were exported from the CVH by using a MySQL query to produce a link-list format, which consists of two columns – column A for the name of the committee and column B for the name of each member – for example, if a committee had ten members then there were ten rows. The linked list format is used in visualization algorithms (tree algorithm for the hierarchy graph and spring-embedding algorithm for the interlock graph) that were coded into the cloud software.

### Data analysis

**Figure 1:**
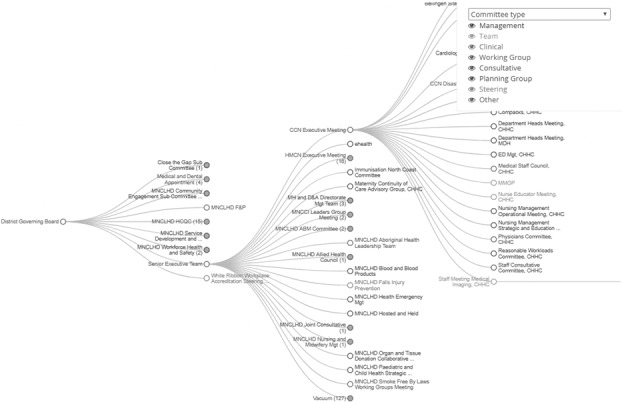
Hierarchical governance structure visualization of committees within the MNCLHD

The data were analyzed using specialist software NetMiner 4.2 (Cyram, 2016; Sofia Pereira and Soares, 2007). The number of links (connections) for a clinician is equal to the number of committee memberships help, which is degree centrality (Butts, 2008), and is visualized as a larger symbol. The same applies for each committee, where the size of the icon is equivalent to more committee members. ToR were analyzed by counting the “headings” of each ToR document. Governance chains were enumerated by counting the number of steps between hierarchically connected committees (see Figure 1), thus providing the degrees of separation measure akin to “six degrees of separation” (Luke and Harris, 2007).

## Results

Because there was an identified lack of clinician engagement, the VotC project aimed to map existing governance structures and to create a technological platform that made clinician voice visible through complex networking techniques. The results of the first year of the VotC project are sociologically significant in that membership of committees is a mechanism to be included in hierarchical decision making, and that interlocking different committees allows for strategic influence in an organization.

### Hierarchy committee mapping

The results of the VotC baseline data collection efforts showed 323 committees in the MNCLHD which were then used to construct the hierarchical governance structure visualization in Figure 1. In its entirety, the governance structure visualization created by data in the CVH is large and nuanced. The most important conceptual element to explain is that committees are linked into a formal decision-making system (see Figure 1).

In the formal hierarchy, where committees report to other committees, it is possible to see how they are connected to the governing board (far left of Figure 1). This reveals a high degree of complexity in organizational architecture. First, the map reveals the 323 committees as points whose knowledge flows through numerous pathways to the governing board. Additionally, it introduces the concept of the governance chain as a shorthand way to represent the fact that interorganizational connections exist in a hierarchical relational structure and that this has important implications for the assessment of clinician voice diffusion into the decision-making structure of the organization, which is discussed further below.

The most stunning finding, however, is that 123 committees could not be found to link with the governing board (Figure 1, at the bottom). These committees were linked into the artificial “vacuum” to signify that the voices from them flow into emptiness due to the absence of a formally stated link to any superordinate committee. This is significant for the effective governance of the organization because of both the time clinicians spend in meetings, and the wage cost of those meetings. For the calculations, each of the 926 people in the database attend 3.4 meetings, each meeting occurs an average of 18 times per year, resulting in 61 meetings per person per year. The frequency of meetings varies across committee function. For example, some committees meet quarterly, representing a network or district level and cover strategic issues. On the other hand, localized team committees meet on a weekly basis while bed management meetings convene most frequently at 260 times per year and are entirely operational in nature. Again, if one assumes 1.5 hours per meeting across 61 meetings multiplied by 926 people then that equals 84,729 person hours. Furthermore, assuming an average AUD$45 per person/hour results in AUD$3.812 M. Therefore, it is reasonable to deduce that meetings are costly and time consuming exercises that should be subjected to measures of efficiency and effectiveness.

### Committee interlock network

**Figure 2:**
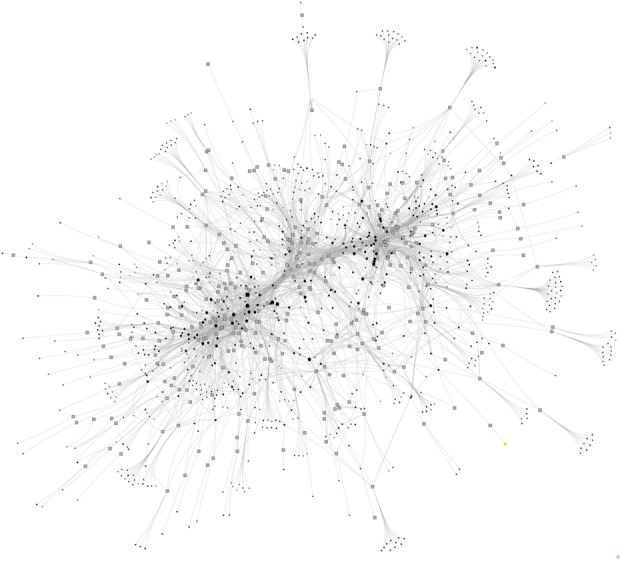
Committee interlock graph for the MNCLHD

Networks offer a complementary view to hierarchies, where, analogous to that of a spider’s web, clinicians may have their views heard through being interconnected to other knowledge brokers. The key point is that individuals may be members of more than one committee, in social network terms this is called an “interlock” (Butts, 2008). The main interlock graph (Figure 2) is constructed from membership data from 323 committees and 926 committee members, which equates to 3,159 links, a vastly different representation to that of the formal links in the Figure 1. The key finding is that all 926 committee members were interlinked into the decision-making structures, this contrasts to the hierarchy, where the committees and committee members of the “vacuum” appear disconnected. This means that clinicians may influence decision-making processes through their connectedness, although whether or not this occurs is another subject for future investigation.

**Figure 3:**
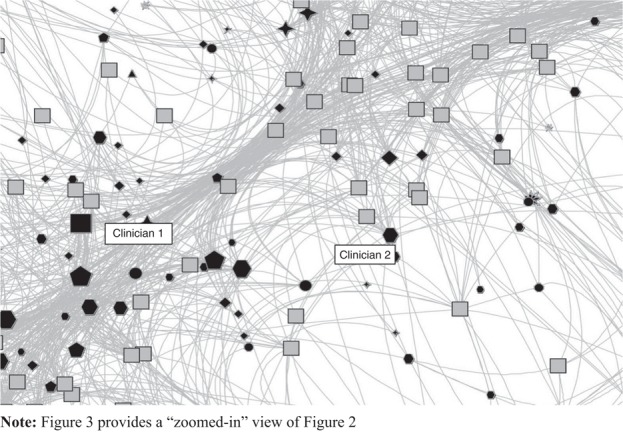
Detailed view of the center of the MNCLHD committee interlock graph

As is evident from Figure 2, the committee interlock data are also extremely difficult to visualize in print, which are discussed later in the development of the CVH. Nevertheless, some general topographical features are evident. The overall structure has a dense core to which a large number of links are generated from the periphery. Clearly, some committees (grey squares) and committee members (various shapes) are more interconnected into the decision-making structures of the MNCLHD. Whether this converts into greater levels of influence, or greater levels of information overload, requires more investigation. However, a key informant noted that most of the frontline clinicians are located at the periphery of the network and often had just one link to a committee. Managers are more connected whilst directors and executives have multiple connections and occupy the core of the interlock network, as can be seen in Figure 3.

As such, Figure 3 shows the examination of different segments of the committee interlock. The committees are grey squares, the members are the different shapes, where each shape represents a different clinician type (medical doctor, nurse, dietitian, etc.). The size of each symbol is equivalent to the number of links for each committee member. For example, clinician 1 has 35 links to committees, whilst clinician 2 has just five links to committees. That some committee members are more connected than others is represented in the average number of links (average *n*=3.4, range *n*=1-43, SD=4.8). That committees differ in the number of members is also evident in the size of the committee symbol (average *n*=10, range *n*=1-100, SD=10). Future analysis can reveal important insights into the relevance of this data for clinicians’ voice. It is also evident that reported data are imprecise with a committee having one member and another committee having 100 members. This is most likely due to poorly structured ToR. However, the data presented herein are the most accurate that are available and give the best estimate to date. Until there is increased transparency of meeting attendance, the quality of data cannot improve. This is one area where the CVH innovation of the VotC project can immediately improve practice as it will streamline the collection of such meeting data thereby yielding more robust estimates. As such, these quality issues are the subject of further research in 2017-2018.

### Audit of ToR

Alongside of the maps of the hierarchy and the interlocks based on links is the importance of the processes of each committee as embedded in their ToR. Of the 323 committees, the results found that just 113 had ToR. In total, 39 (35 percent) were sampled and it was found that they were highly variable in there heading structure (Table I). From these it is evident that none of the committees were reviewed or evaluated, there is no information on how agendas are generated, nor is there information on how the minutes are processed. These are key issues of transparency and accountability that speak to a lack of value given to clinicians’ issues, and the lack of feedback to clinicians about the resolution of their issues.

### Governance chain analysis

Both the hierarchy and the interlock graphs reveal the complexity of assessing the influence of clinicians’ voice in decision-making structures. Each committee is a number of steps away from the District Governing Board (Figure 1), or the degrees of separation, which is an important measure of social distance and social influence in health research (Valente *et al.*, 2008; Fowler and Christakis, 2008). For example, the District Health Care Quality Committee is one degree from the governing board. In contrast, the Standards Working Party is three degrees removed. Within this complexity, what happens to clinicians’ voice through the steps from one committee to the next, as ideas flow upstream to the governing board? Whether the influence of clinician voice increases the closer a committee is to the governing board will be the focus of the next phase of the VotC project.

**Figure 4:**
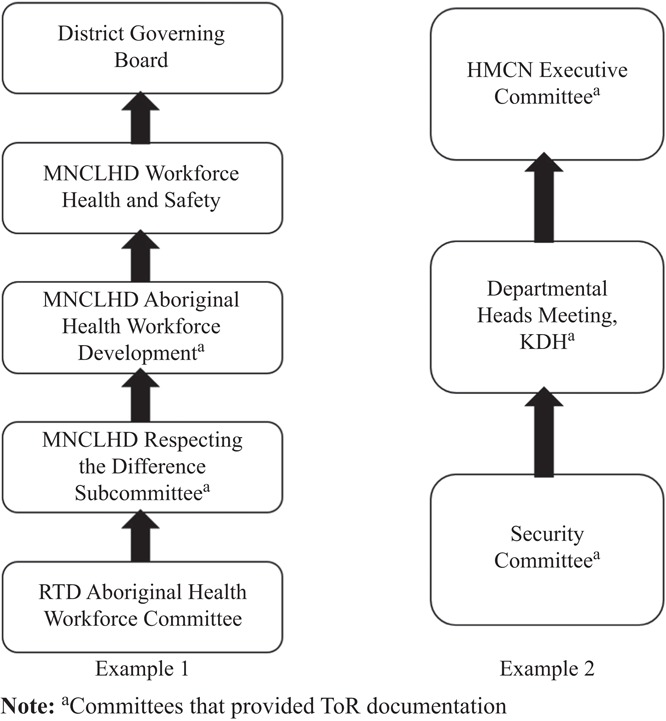
Two examples of committee reporting structures

Furthermore, a key task of the VotC project is to assess how clinicians’ voice moves from a subordinate committee to a superordinate committee in terms of their issues being “escalated” to committees with higher decision-making authority. Because both the hierarchy link data and the ToR were collected it is possible to assess the diffusion of clinician voice. The principle is that voice should be enabled to flow upstream from subordinate to superordinate committees (see examples provide in Figure 4).

Example 1 in Figure 4 shows that not all committees provided ToR documentation. In contrast, example 2 shows that each committee provided ToR, and thus this alignment can be assessed for how clinicians’ voice travels upstream. The key insight from Figure 4 is that clinicians’ voice travels through many points before – possibly – reaching the governing board. However, whether the frontline clinicians’ voice reaches the governance board cannot be assessed due to the lack of ToR, the absence of minutes from any of the committees, and the absence of any documentation of actions arising from each committee. These pieces are necessary to see if clinicians are given time on the agenda, which should be included in the ToR, to raise frontline issues, if those issues are present in the minutes and whether issues were escalated upstream to the next committee. These quality issues are to be addressed in further work.

In summarizing the key points of the results, the evidence shows that many committees do not, in fact, connect to decision-making structures. Instead, these committees are linked to an artificial “vacuum” which suggests that the voices within the committee may not be heard by organizational decision-makers. However, while some committees may not have had hierarchical links to decision-making structures, all committee members were connected to decision-making structures via the interlocks. This suggests that, even if not directly, clinicians may have influence over decision making through levels of connectedness. Furthermore, there is evidence that connectedness varies greatly across members. Also, and perhaps one result that can yield immediate practical change, was the discovery that only 35 percent of the committees even had ToR. The lack of ToR documentation suggests that such committees may be susceptible to inefficient feedback loops wherein the clinician’s concerns may not have been passed upward to decision-makers nor may they hear about the resolution of their original concerns.

## Unresolved questions and lessons for the field

The VotC project, to date, has exceeded expectations and been received with much optimism with work to continue in 2017-2018. The purpose of the first year (2016) was to determine and describe the structural location of clinicians’ voice in organizational decision-making processes in a health system in Australia. The project provided a baseline illustration of the current committee activity and impediments to the VotC not being heard by those in decision-making power in the organization. In that way, the project has been quite successful.

One of the more prominent future opportunities involves further exploration into whether restructuring the linkages – like restructuring road networks – results in more effective communication of clinician issues to executive decision-makers. Moving forward, the next phase of the VotC project will include a committee audit, review and redesign strategy: an audit of all committee charters/ToR (American Hospital Association, 2008), the collection of interview and survey data that further captures the clinician perspective, statistical modeling of social network metrics with clinician engagement survey response, and further develop the CVH technology. Additionally, the CVH technology will be implemented on a larger scale that traverses well beyond the borders of the MNCLHD system. Lastly, the project will be replicated in a different cultural setting and one with a structurally dissimilar health system, namely, the USA.

Finally, translating this project into practice involves deploying the CVH on a centralized computer system in combination with an education and training program. The transferability and scalability are underpinned by a committee system common to all local health districts using the same database variables, centralized server, and web-based interface, further permitting individual log into an engaging and interactive committee network map. By drawing on the perspectives of system-wide communication, individual clinician voice in the system, team-level information, and network influence, the VotC project and CVH innovation explicitly capture committee purpose which empowers clinicians’ voice and enables the further observation of their input creating organizational change. This outcome directly tackles the concerns shared by Cohn (2015) and Sears (2011) who identified listening to clinician perspective and implementing it into decision making as tied to engagement. In conclusion, through the VotC project and CVH, clinicians will be able to see all organizational committees and how they are linked into the formal hierarchical structure of the system. With implementation, the CVH unlocks the potential for clinicians to communicate clinical issues to other committees, visibly see their voice (and other clinician voices) in the system through the committee interlock, observe their committees in the system with the ability to access and edit information, and witness how influence can be leveraged through networked governance.

## Figures and Tables

**Table I:** Sections of terms of reference

Heading	Sum	%
Membership/Orientation	39	100
Title	35	90
Purpose	32	82
Badged	30	77
Terms of Reference	29	74
Quorum	27	69
Objectives	25	64
Meeting Frequency	25	64
Reporting/Delegations	25	64
Agenda/Minutes/Meeting Papers	25	64
Chairperson	22	56
Evaluation & Review	21	54
Authority	20	51
Secretariat/Support	17	44
Meeting Procedures	13	33
Meeting Time/Venue	12	31
Membership Variables/Alternate	9	23
Signature/Authorisation	9	23
Role	7	18
Standing Items	7	18
Responsibility	3	8
Venue	3	8
Invitees	3	8
Meeting Duration	3	8
Accountability	3	8
Reports for Tabling	3	8
Background	2	5
Introduction	2	5
Voting Rights	2	5
Co-chair responsibilities	2	5
Minutes	2	5
Conflict of Interest	2	5

**Notes:** The following headings had one reference each and represented 3 percent of the total, respectively: “aim,” “program,” “strategic context,” “guiding principles,” “key performance indicators,” “roles and responsibilities of committee members,” “funding,” “appointment of co-chairs,” “scope of representation,” “decision making,” “working groups,” “apologies,” “linkages,” “budget,” “sub-working groups,” “stakeholders,” “confidentiality,” and “media”
